# Medical image captioning via generative pretrained transformers

**DOI:** 10.1038/s41598-023-31223-5

**Published:** 2023-03-13

**Authors:** Alexander Selivanov, Oleg Y. Rogov, Daniil Chesakov, Artem Shelmanov, Irina Fedulova, Dmitry V. Dylov

**Affiliations:** 1 Skolkovo Institute of Science and Technology, Bolshoy blvd., 30/1, Moscow, 121205 Russia; 2 Philips (Russia), Skolkovo Technopark 42, Building 1, Bolshoi Boulevard, Moscow, 121205 Russia; 3 AIRI, Kutuzovsky Ave, 32 bld. 1, Moscow, 121170 Russia

**Keywords:** Radiography, Computer science

## Abstract

The proposed model for automatic clinical image caption generation combines the analysis of radiological scans with structured patient information from the textual records. It uses two language models, the Show-Attend-Tell and the GPT-3, to generate comprehensive and descriptive radiology records. The generated textual summary contains essential information about pathologies found, their location, along with the 2D heatmaps that localize each pathology on the scans. The model has been tested on two medical datasets, the Open-I, MIMIC-CXR, and the general-purpose MS-COCO, and the results measured with natural language assessment metrics demonstrated its efficient applicability to chest X-ray image captioning.

## Introduction

Medical imaging is indispensable in the current diagnostic workflows. Out of the plethora of existing imaging modalities, X-ray remains one of the most widely-used visualization methods in many hospitals around the world, because it is inexpensive and easily accessible^1^. Analyzing and interpreting X-ray images is especially crucial for diagnosing and monitoring a wide range of lung diseases, including pneumonia^2^, pneumothorax^3^, and COVID-19 complications^4^.

Today, the generation of a free-text description based on clinical radiography results has become a convenient tool in clinical practice^5^. Having to study approximately 100 X-rays daily^5^, radiologists are overloaded by the necessity to report their observations in writing, a tedious and time-consuming task that requires a deep domain-specific knowledge. The typical manual annotation overload can lead to several problems, such as missed findings, inconsistent quantification, and delay of a patient’s stay in the hospital, which brings increased costs for the treatment. Among all, the qualification of radiologists as far as the correct diagnosis establishing should be stated as major problems.

In the COVID-19 era, there is a higher need for robust image captioning^5–7^ framework. Thus, many healthcare systems outsource the medical image analysis task. Automatic generation of chest X-ray medical reports using deep learning can assist and accelerate the diagnosis establishing process followed by clinicians. Providing automated support for this task has the potential to ease clinical workflows and improve both care quality and standardization. For that, we propose to adapt powerful models from non-medical domain.

### Medical background

Radiology is the medical discipline that uses medical imaging to diagnose and treat diseases. Today, radiology actively implements new artificial intelligence approaches^8–10^. There are three types of radiologists—diagnostic radiologists, interventional radiologists and radiation oncologists. They all use medical imaging procedures such as X-rays, computed tomography (CT), magnetic resonance imaging (MRI), nuclear medicine, positron emission tomography (PET) and ultrasound. Diagnostic radiologists interpret and report on images resulted from imaging procedures, diagnose the cause of patient’s symptoms, recommend treatment and offer additional clinical tests. They specialize on different parts of human body—breast imaging (mammograms), cardiovascular radiology (heart and circulatory system), chest radiology (heart and lungs), gastrointestinal radiology (stomach, intestines and abdomen), etc. Interventional radiologists use radiology images to perform clinical procedures with minimally invasive techniques. They are often involved in treating cancer, heart diseases, stroke, blockages in the arteries and veins, fibroids in the uterus, back pains, liver and kidney problems.

### Technical background

Because image captioning is a multimodal problem, it draws a significant attention of both computer vision and natural language processing communities. The latest surveys in the medical image captioning task^5,11^ offer a detailed description of domain knowledge from radiology and deep learning. The first architectures to address this problem were CNN-RNN models from^12,13^. However, the latter shows satisfactory results only on the single-pathology tasks.

With the new concept of attention approach^14^, more papers have begun to use visual attention^15–17^, being the first to use attention on medical images. The authors of^15^ presented a model that can fix its attention on salient objects while generating the corresponding words in the output sequence. Shortly after the visual-attention concept was exposed, text-attention was introduced by authors of TieNet—a framework that generates natural reports^18–20^ for the Chest-Xray dataset^21^. They used both semantic and visual attention, that allowed them to get high natural language generation (NLG) metrics on medical datasets. It was trained for solving several tasks such as classification, localization, and text generation. It used a non-hierarchical CNN-LSTM^22^ approach together with the attention to semantic and visual features, as it allowed to overperform the current state-of-the-art results. In the^23^, bone fracture X-ray reports were generated by identifying image features and filling text templates. The authors of^20^ suggested a multi-task framework, that can both predict tags and generate texts using co-attention. This model is still not sufficient for producing accurate diagnosis from X-rays as the produced texts still contained repeated sentences due to a lack of contextual coherence in the hierarchical models. The authors of^24^ took advantage of a sentence-level attention mechanism in a late fusion fashion. They took advantage of the multi-view images from both frontal and lateral view angles from the Open-I dataset^25^.

The authors of^26^ proposed to utilize a pre-constructed knowledge graph embedding module (extracted from the Open-I images using Chexnet models^27^) on multiple disease findings to assist the report generation process. The authors of^28^ exposed an anomaly detection method for detecting abnormalities on chest X-rays with deep perceptual autoencoders. The authors of^29^ first generated topics for sentences using reinforcement learning (RL) followed by the word decoder sequence generation from the topic with attention to the original images. RL was used for tuning to optimize readability. We solve this problem in a simpler method without losing in quality. To extract topics, we use the NegBio labeller^21,30^, which provides topics from clinical reports. We add these topics to the beginning of the medical report, for our model to understand where exactly the text should be generated.

The work^31^  focuses on reporting abnormal findings on radiology images. The proposed method learns conditional visual-semantic embeddings in radiology images; and the reports are further used to measure the similarity between the image regions and the medical reports. This by optimizing a triplet ranking loss. The authors of^32^ developed an algorithm that learns a description of findings from images and uses their pattern of occurrences to retrieve and customize similar reports from a large report database. The work in^33^ proposed a Contrast Induced Attention Network (CIA-Net), using contrastive learning on the aligned positive and negative samples for the disease localization on the chest X-ray images. The work in^34^ studies the cross-domain performance, agreement between models, and model representations for X-rays diagnostic prediction tasks. The authors test for concept similarity by regularizing a network to group tasks across multiple datasets together and observe variation across the tasks. The model in^22^ generates a short textual summary with essential information on the found pathologies along with their location and severity. The model is trained on only 2% of the MIMIC-CXR dataset, and generates short reports. Although, in this work, we train on the whole MIMIC-CXR and generate a full-text report.

The authors of^35–39^ attempted to use transformer-based models as decoders in the image captioning domain^22^. The work^38^ affirmed to have generated radiology reports through the custom transformer with additional memory-driven unit. Another model was introduced in^39^ where encoder detects regions of interest via a bottom-up attention module and extracts top-down visual features. In this study, the decoder is presented as a custom transformer. For example, the paper in^36^ proposes an approach called “pseudo self-attention”. Its main idea is to incorporate the conditioning input as a pseudo history to a pretrained transformer. They add a new key and value weights in the self-attention module to be projected onto the decoder’s self-attention space, while^37^ focuses on visual and weighted semantic features.

### Contributions

The contributions of this paper are the following:

- We introduce a new architecture for image captioning, based on a combination of two language models with image-attention (SAT) and text-attention (GPT-3), outperforming current state-of-the-art models
- We introduce a new preprocessing pipeline for radiology reports that allows to get higher NLG metrics
- We perform extensive experiments to show the capability of the proposed method
- Finally, we contribute to deep learning community by training two language models on a large dataset MIMIC-CXR

The rest of the paper is organized as follows: section “Methods” describes the architecture of two language models separately, section “Proposed architecture” provides the description of the proposed approach, section “Experiments” describes the data and the computing, the last sections compare the results and conclude the paper.

## Methods

### Show attend and tell

Show Attend and Tell (SAT)^15^ is an attention-based image caption generation neural net. An attention-based technique allows to get well interpretable results, which can be utilized by radiologist to ensure their findings on X-ray. By including attention, the module gives the advantage to visualize where exactly the model ‘sees’ the specific pathology. SAT consists of three blocks: Encoder, Attention module, and Decoder. It takes an image, encodes it, attends each part of the image, and generates an *L*-length caption ${\textbf{z}}$, an encoded sequence of words from the *W*-length vocabulary:

1 $$\begin{aligned} {\textbf{z}}= \left\{ {\textbf{z}}_1, \ldots , {\textbf{z}}_{L} \right\} ,\quad {\textbf{z}}_i \in {\mathbb {R}}^{W_{SAT}} \end{aligned}$$

#### Encoder

Encoder is a convolutional neural network (CNN). It encodes an image and outputs a set of *C* vectors, each of which is a *D*-dimensional representation of the image corresponding part:

2 $$\begin{aligned} {\textbf{a}}= \left\{ {\textbf{a}}_1, \ldots , {\textbf{a}}_C \right\} ,\quad {\textbf{a}}_i \in {\mathbb {R}}^{D \times D} \end{aligned}$$

Here, *C* represents the number of channels in the output of the encoder. It depends on the used type of the encoder: 1024 for DenseNet-121^40^, 512 for VGG-16^41^, 2048 for InceptionV3^42^ and ResNet-101^43^. *D* is a configurable parameter representing the encoded vectors size. Features are extracted from the lower convolutional layer prior to the fully connected layers, and are being passed through the Adaptive Average Pooling layer. This allows the decoder to selectively focus on certain parts of an image by selecting a subset of all the feature vectors.

#### Decoder with attention module

The decoder is implemented as an LSTM neural network^44^. It produces a caption by generating one word at every time step conditioned by the attention (context) vector, the previous hidden state and the previously generated words. The LSTM can be represented as the following set of equations:

3 $$\begin{aligned} \begin{pmatrix} {\textbf{i}}_t \\ {\textbf{f}}_t \\ {\textbf{o}}_t \\ {\textbf{g}}_t \\ \end{pmatrix}&= \begin{pmatrix} \sigma \\ \sigma \\ \sigma \\ \tanh \\ \end{pmatrix} T_{D+m+n, n} \begin{pmatrix} {\textbf{Ez}}_{t-1}\\ {\textbf{h}}_{t-1}\\ \hat{{\textbf{a}}_t}\\ \end{pmatrix} \end{aligned}$$

4 $$\begin{aligned} {\textbf{c}}_t&= {\textbf{f}}_t \odot {\textbf{c}}_{t-1} + {\textbf{i}}_t \odot {\textbf{g}}_t \end{aligned}$$

5 $$\begin{aligned} {\textbf{h}}_t&= {\textbf{o}}_t \odot \tanh ({\textbf{c}}_{t}). \end{aligned}$$

**Figure 1:**
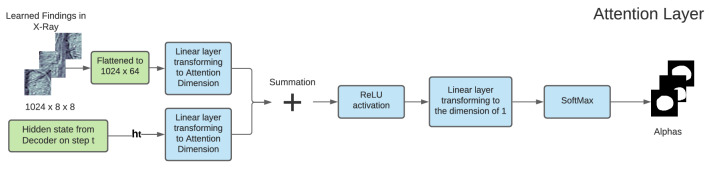
Attention module used in SAT.

Vectors ${\textbf{i}}_t$, ${\textbf{f}}_t$, ${\textbf{c}}_t$, ${\textbf{o}}_t$, ${\textbf{h}}_t$ represent the input/update gate activation vector, forgetting gate activation vector, memory or cell state vector, while outputting gate activation vector and hidden state of the LSTM respectively. $T_{s,t}$ is an affine transformation, such that ${\mathbb {R}}^{s} \rightarrow {\mathbb {R}}^{t}$ with non-zero bias. *m* denotes the embedding dimension, while *n* represents LSTM dimension. $\sigma$ and $\odot$ stand for the sigmoid activation function and element-wise multiplication, respectively. ${\textbf{E}}\in {\mathbb {R}}^{m\times L}$ is an embedding matrix. The vector ${\hat{\textbf{a}}} \in {\mathbb {R}}^{D}$ holds the visual information from a particular input location of the image at time *t*. Thus, ${\hat{\textbf{a}}}$ called context vector. Attention is a function $\phi$, that computes context vector ${\hat{\textbf{a}}}_t$ from the encoded vectors $\textbf{a}_i$ (2), produced by the encoder. The attention module generates a positive number $\alpha _i$ for each location *i* on the image. This number can be interpreted as the relative importance to give to the location *i*, among others. Attention module is implemented as a multi-layer perceptron (MLP) with a softmax activation function, conditioned at the previous hidden state $h_{t-1}$ (5) of the LSTM. The attention module is depicted in Fig. 1. The set of linear layers in MLP is denoted as a function $f_{\text{ att }}$. The weights $\alpha _{ti}$ are computed using the following equations:

6 $$\begin{aligned} e_{ti}&= f_{\text{ att }} ({\textbf{a}}_i, {\textbf{h}}_{t-1}) \end{aligned}$$

7 $$\begin{aligned} \alpha _{ti}&= \frac{\exp (e_{ti})}{\sum _{p=1}^C \exp (e_{tp})} \end{aligned}$$

The sum of weights $\alpha _{ti}$ (7) should be equal to 1 $\sum _{i = 1}^{C} \alpha _{ti} = 1$. The context vector ${\hat{a}}_t$ is computed by the *attention function* $\phi$ with the set of encoded vectors ${\textbf{a}}$ (2) and their corresponding weights $\alpha _{ti}$ (7) as inputs: ${\hat{\textbf{a}}}_t = \phi \left( \left\{ {\textbf{a}}_i \right\} , \left\{ \alpha _{ti} \right\} \right)$. According to the original paper function, $\phi$ can be either ‘soft’ or ‘hard’ attention. Due to specific task of medical image caption, function $\phi$ was chosen to be the ‘soft’ attention, as it allows model to focus more on some specific parts of X-rays from others and to detect pathologies and major organs such as heart, lung etc. It is named as a ‘deterministic soft attention’ and recognized as a weighted sum : $\phi \left( \left\{ {\textbf{a}}_i \right\} , \left\{ \alpha _{ti} \right\} \right) = \sum _i^C \alpha _i {\textbf{a}}_i$. Hence, context vector can be computed as:

8 $$\begin{aligned} {\hat{\textbf{a}}}_t = \sum _i^C \alpha _i {\textbf{a}}_{ti} \end{aligned}$$

The initial memory state and hidden state of the LSTM are initialized with two separate multi-layer perceptrons ($\text {init-c}$ and $\text {init-h}$) with the encoded vectors ${\textbf{a}}_i$ (2) for a faster convergence:

9 $$\begin{aligned} {\textbf{c}}_0&= f_{\text {init-c}} \left( \frac{1}{C} \sum _i^C {\textbf{a}}_i \right) \end{aligned}$$

10 $$\begin{aligned} {\textbf{h}}_0&= f_{\text {init-h}} \left( \frac{1}{C} \sum _i^C {\textbf{a}}_i\right) \end{aligned}$$

To compute the output of LSTM representing a probabilities vector the next word, a ‘deep output layer’^44^ was used. It looks both on the LSTM state ${\textbf{h}}_t$ (5), on context vector ${\hat{\textbf{a}}}_t$ (8) and the one previous word ${\textbf{z}}_{t-1}$ (2):

11 $$\begin{aligned} P({\textbf{z}}_t | {\hat{\textbf{a}}}_t, {\textbf{z}}_{t-1}) = softmax(\textbf{L}_o(\textbf{L}_h\textbf{h}_t+ \textbf{L}_a {\hat{\textbf{a}}}_t + \textbf{E}\textbf{z}_{t-1})) \end{aligned}$$

where $\textbf{L}_o\in {\mathbb {R}}^{W\times m}$, $\textbf{L}_h\in {\mathbb {R}}^{m\times n}$, $\textbf{L}_a\in {\mathbb {R}}^{m\times D}$, and $\textbf{E}\in {\mathbb {R}}^{m\times L}$ represent the embedding matrix.

The authors in^15^ suggest to use the ‘doubly stochastic attention’, where $\sum _t \alpha _{ti} \approx 1$. This can be interpreted as encouraging the model to pay equal attention to every part of the image. Yet, this method is not relevant for X-rays, as each part of the chest is almost at the same position from image to image. If the model learned, e.g., that heart is in its specific position, a model does not have to search for the heart somewhere else. The model is trained in an end-to-end manner by minimizing the cross-entropy loss $L_{CE}$ between vector with a softmaxed distribution probability of next word and true caption as $L_{CE} = -\log (P({\textbf {z}}|{\textbf {a}}))$.

### Generative pretrained transformer

Generative Pretrained Transformer (GPT-3)^45^ is a large transformer-based language model with $1.75 \times 10^{11}$ parameters, trained on 570 GB of text. GPT-3 can be used to generate realistic continuations texts from the arbitrary domain. Basically, GPT-3 is a transformer that can look at a part of the sentence and predict the next word, thus being a language model. The original transformer^46^ is made up of encoder stack and decoder stack, in which encoders and decoders stacked upon each other. Whereas GPT-3 is built using just decoder blocks. One decoder block consists of Masked Self-Attention layer and Feed-Forward neural network. It is called Masked as it pays attention only to previous inputs. The input should be encoded prior to going into the decoder block. In transformers and in the GPT-3 particularly, there are two subsequent encodings: Byte Pair Token Encoding and Positional Encoding. Byte Pair Encoding (BPE) is a simple data compression technique that iteratively replaces the most frequent pair of bytes in a sequence with a single, unused byte. The algorithm compresses data by finding the most frequently occurring pairs of adjacent subtokens in the data and replacing all instances of the pair with a single subword. The algorithm repeats this process until no further compression is possible. Such tokenization avoids adding a special $\texttt {<unk>}$ token to the vocabulary, as now all words can be encoded and obtained by combination of subwords from the vocabulary.

## Proposed architecture

We introduce two architectures for X-ray image captioning. The overall goal of our approach is to improve the quality of Encoder-Decoder generated clinical records by using the GPT-3 language model. The suggested model consists of two parts: the Encoder, Decoder (LSTM) with an attention module and GPT-3. While Encoder with LSTM detects pathologies and indicates zones of higher attention demand, the GPT-3 takes it as input and writes a comprehensive medical report. There are two possible approaches for this task.

**Figure 2:**
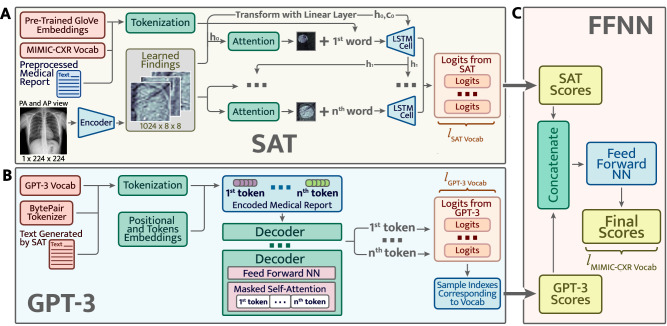
The first approach. Learn the joint distribution of two models.

**Approach 1** The first method consists in forcing the models to learn a joint word distribution. Within this method (Fig. 2), both models **A** and **B** output scores for the next word in a sentence. Afterwards, due to concatenating these scores and pushing them through the feed-forward neural net **C**, we get the final scores for subsequent word. Whilst the disadvantage of this approach is the following: the GPT-3 model has its own vocabulary built by the byte pair tokenizer. This vocabulary is different from the one used by the SAT model. We need to take from continuous GPT-3 distribution separate scores corresponding to the words present in the Show Attend and Tell vocabulary. This turns continuous distribution from the GPT-3 into discrete and hence, while we do not use all the potential generation power from the GPT-3.

**Figure 3:**
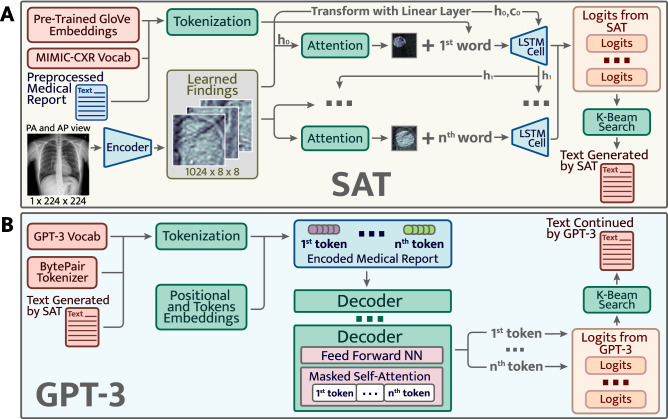
The second approach. Pretrained GPT-3 (**B**) continues text generated by SAT (**A**).

**Approach 2** The Approach 2 is shown in Fig. 3 and is based on stacked **A** and **B** models. Show Attend and Tell **A** gets an image as an input and generates a report based on the data found on X-ray with an Attention module. It learns where to focus and gives a seed for the GPT-3 **B** to continue generating text. The GPT-3 was fine-tuned on MIMIC-CXR in self-supervised manner using the Huggingface framework^47^. It learns to predict the next word in the text. The GPT-3 continues the report outputed by SAT and generates a detailed and complete clinical report based on pathologies found by SAT. Such an approach is better for the GPT-3 as it gets more context as input (from SAT) than in the first approach. Thus, the second approach performs better, and was hence chosen as the main architecture.

### First language model

The first part of the suggested model is realized as the Show Attend and Tell model (SAT), the encoder to encode the image, and the LSTM for decoding into sequence. The encoder encodes the input image with 3 or 1 color channels into a smaller image with ‘learned’ channels. The resulted encoded images can be interpreted as a summary representation of findings in the X-ray (Eq. 2). Those encoders, pretrained on the ImageNet^48^, are not suitable for the medical image caption task, as medical images do not have typical objects from the natural domain. Thus, the DenseNet-121 from^49^ pretrained on the MIMIC-CXR dataset was taken. It was trained for the classification task on 18 labels : Atelectasis, Consolidation, Infiltration, Pneumothorax, Edema, Emphysema, Fibrosis, Effusion, Pneumonia, Pleural Thickening, Cardiomegaly, Nodule, Mass, Hernia, Lung Lesion, Fracture, Lung Opacity, and Enlarged Cardiomediastinum. Hence, the last classification layer was removed and features from the last convolutional layer were taken. These features were passed through the Adaptive Average Pooling layer. They can be represented by the tensor with the following dimensions: ($batch size \times C, D, D$) (Eq. 2). *C* stands for the number of channels or how many different image regions to consider. *D* implies the dimension of the image encoded region. Furthermore, the fine-tune method for encoder was added. It enables or disables the calculation of gradients for the encoder’s parameters through the last layers. Then, at every time step, the decoder with the attention module observes the encoded small images with findings and generates a caption word by word. The Encoder output is received and flattened to dimensions ($batch size, C, D \times D$). Since captions are padded with a special token $\texttt {<pad>}$, captions are sorted by decreasing lengths and at every time-step of generating a word, an effective batch size is computed in order not to process the $\texttt {<pad>}$ token.

The Show Attend and Tell model was trained using the Teacher-Forcing method while at each step the input to the model was the ground truth word on this step and not the previous generated word. As a result, we can consider the SAT as a language model ${\textbf {A}}$. It gets a tokenized text of length *m*, an image as input and outputs a vector of probabilities for the next word at each time step *t*:

12 $$\begin{aligned} \begin{aligned} {\textbf {A}} : \text {text,image} \rightarrow P_1(\textbf{z}^t | \text {true words} = \textbf{z}^{\langle 1\rangle }\textbf{z}^{\langle 2\rangle }\dots \textbf{z}^{\langle t-1\rangle }, \text { image}), \\ t \in \{2, \dots m, \dots L\}, \\ P_1 \in {\mathbb {R}}^{m \times W_{SAT}} \end{aligned} \end{aligned}$$

where *W* is the SAT vocabulary size and L is the length of generated report (Eq. 1). Where $P_1$ is computed as it is shown in the Eq. (11).

Over the training process, the LSTM outputs a word with a maximum probability after the softmax layer. Similarly to^50^, we applied the K-Beam search, but only in the inference stage.

### Second language model

The second part of the architecture proposed is the GPT-3. The GPT-3 is built from decoder blocks using the transformer architecture. At the same time, the decoder block consists of masked self-attention and a feed-forward neural network (FFNN). The output yields the token probabilities, i.e., logits. The GPT-3 was pretrained separately on the MIMIC-CXR dataset and was then fine-tuned together with the SAT to enhance clinical reports.

We put a special token $\texttt {<start>}$ at the end of the text generated by the SAT allowing the GPT-3 to understand where to start the generation process. We also used the K-Beam search after the GPT-3 generation and took the second best sentence from the output as a continuation. The pretrained GPT-3 performs as a separate language model ${\textbf {B}}$ and generates good records based on the input text or tags. The GPT-3 generates report till the moment when it generates the special token $\texttt {<|endoftext|>}$. We denote the length of the GPT-3 generated text as *l*

13 $$\begin{aligned} \begin{aligned} {\textbf {B}} : \text {text} \rightarrow P_2(\textbf{z}^t | \text {true words} = \textbf{z}^{<1>}\dots \textbf{z}^{<L>}<\textbf{s}>), \quad t \in \{L+1, \dots L + l\}, \\ \end{aligned} \end{aligned}$$

### Combination of two language models

We use a combination of two models placing them sequentially: the SAT model extracts visual features from the image and allows us to focus on its specific parts. The GPT-3 provides good and comprehensive text, based on what is found by the first model. Thus, the predictions from the first model improve those of the second language model.

### Evaluation metrics

The common evaluation metrics used for image captioning are : bilingual evaluation understudy (BLEU)^51^, recall-oriented understudy for gisting evaluation (ROUGE)^52^, metric for evaluation of translation with explicit ordering (METEOR)^53^, consensus-based image description evaluation (CIDEr)^54^, and semantic propositional image caption evaluation (SPICE)^55^. The Microsoft Common Objects in Context^56^ provides the kit with implementation of these metrics for the image caption task.

## Experiments

### Datasets

For training and evaluation of medical image captioning, we use three publicly available datasets. Two of them are medical images datasets and the third one is a general-purpose one.

**MIMIC-CXR** The MIMIC Chest X-ray (MIMIC-CXR)^57^ dataset is a large publicly available dataset of chest radiographs in DICOM format with free-text radiology reports. This dataset consists of 377,110 images corresponding to 227,835 radiographic studies performed at the Beth Israel Deaconess Medical Center in Boston, MA.

**Open-I** The Indiana University Chest X-ray Collection (IU X-ray)^25^ contains radiology reports associated with X-ray images. This dataset contains 7470 image-report pairs. All the reports enclose the following sections: impression, findings, tags, comparison, and indication. We use the concatenation of impression and findings as the target captions.

**MSCOCO** Microsoft Common Objects in Context dataset (MS COCO dataset)^58^ is large-scale non-medical dataset for scene understanding. The dataset is commonly used for training and benchmark object detection, segmentation, and captioning algorithms.

### Image preprocessing

Hierarchical Data Format (HDF5)^59^ dataset was used to store all images. X-rays are in gray-scale and have one channel. To process them with the pre-trained CNN DenseNet-121, we used 1 channel image. Each image was resized to the size of $224 \times 224$ pixels, normalized to the range from 0 to 1, and converted to the float32 type and stored in the HDF5 dataset.

### Image captions pre-processing

Following the logic in^60^, a medical report is considered as a concatenation of Impression and Findings sections, if both of these sections are empty, this report was excluded. This resulted in 360,666 DICOMs with reports for the MIMIC-CXR dataset. The text records are pre-processed by converting all tokens to lowercase, removing all non-alphanumerical tokens. For our experiments we used 75% of data for training, 24.75 % for validation and 0.25% for testing.

The MIMIC-CXR database was used to access metadata and labels derived from free-text radiology reports. These labels were extracted using the NegBio tool^21,30^ that outputs one of 14 pathologies along with their severity and (or) absence. To generate more accurate reports, we added the extracted labels to the beginning of the report. This allows language models to know the summary of the report for a more precise description generation.

We additionally formed the abbreviations dictionary of 150$+$ words from the Unified Medical Language System (UMLS)^61^. We also extended our dictionary size with several commonly used medical terms from the Medical Concept Annotation Tool^62^.

### Training of the neural network

The pipeline is implemented using PyTorch. Experiments were conducted on a server running the Ubuntu 16.04 (32 GB RAM). All models were trained with NVIDIA Tesla V100 GPU (32 GB RAM). In all experiments, we use a 5-fold cross-validation and reported the mean performance. The SAT was trained for 70 epochs with the batch size of 16, embedding dimension of 100, attention and decoder dimension of 512, dropout value 0.1. The encoder and decoder learning rates were $4\times 10^{-7}$ and $3\times 10^{-7}$, respectively. The Cross Entropy loss was used for training. The best model is chosen according to the highest geometric mean of BLEU-n, as it is done in other works^63^. SAT was trained in Teacher-Forcing technique, while the Greedy approach is used for counting metrics. The GPT-3 small was fine-tuned with the MIMIC-CXR dataset for 30 epochs with batch size of 4, learning rate of $5\times 10^{-5}$, the Adam epsilon of $1\times 10^{-8}$, where the block size equals 1024, with clipping gradients, which are bigger than 1.0. It was fine-tuned in a self-supervised manner as a language model. No data augmentation was applied.

## Results and discussion

### Quantitative results

The quantitative results for the baseline models, preceding works, and our models are presented in Table 1. The models were evaluated on the most common Open-I dataset, as well as on the big and rarely reported data from the MIMIC-CXR with free-text radiology reports. We implemented the most commonly used metrics for evaluation—BLEU-n, CIDEr and ROUGE_L. The proposed approach outperforms the existing models in terms of the NLG metrics—BLEU-n, CIDEr and ROUGE. BLEU-n measures the accuracy, ROUGE_L measures the recall of the generated report while CIDEr helps estimate the ability of the model to capture context information in the ground truth report. The higher the metrics values, the better the performance of the model.

**Figure 4:**
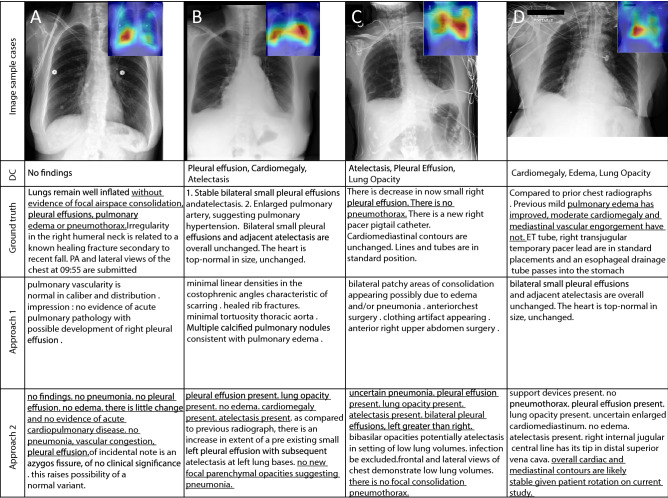
Image sample cases with the disease classes (DC) along with original (ground truth) and generated reports by the proposed SAT + GPT-3 model implemented as in Approach 1 and 2, respectively. Insets in the upper corners of the original images feature localization heatmaps. Heatmaps are generated using Matplotlib v.3.7.0^64^.

We additionally illustrated the performance of our model in Fig. 4 containing 4 original X-ray images from the MIMIC-CXR dataset, the ground truth expert label, and the model predictions (Approaches 1 & 2). We manually underlined the similarities and identical diagnoses in texts to guide the eye. Table 2 presents the measured clinical efficacy (CE) metrics on the MIMIC-CXR dataset for the baseline models and our proposed Approaches 1 and 2. The metrics are calculated by comparing the critical radiology terminology extracted from the generated and the reference reports.

**Table 1:** Reported mean performance using word-overlap metrics for two medical radiology datasets and one non-medical for general purpose. Models labelled with $\dagger$ stand for the models we implemented and trained with the preprocessed MIMIC-CXR data. Other results are cited from the original papers. BLUE-n denotes the BLEU score that uses up to n-grams. The best performance in each configuration is in bold.

	Model			CIDEr	ROUGE_L	BLEU-1	BLEU-2	BLEU-3	BLEU-4
MIMIC-CXR	S &T^12^			0.886	0.300	0.307	0.201	0.137	0.093
	Original SAT^15^			0.967	0.288	0.318	0.205	0.137	0.093
	TieNet^19^			1.004	0.296	0.332	0.212	0.142	0.095
	NLG^29^			1.153	0.307	0.352	0.223	0.153	0.104
	SAT$\dagger$			1.986	0.478	0.634	0.549	0.451	0.383
	Approach 1			1.974	0.477	0.622	0.573	0.497	0.401
	**Approach 2**			**1.989**	**0.480**	**0.725**	**0.626**	**0.505**	**0.418**
Open-I	Co-Attention^60^			0.327	0.447	0.517	0.386	**0.306**	**0.247**
	TieNet^19^			–	0.311	0.330	0.194	0.124	0.081
	CNN-RNN^12^			0.111	0.267	0.316	0.211	0.140	0.095
	LRCN^65^			0.190	0.278	0.369	0.229	0.149	0.138
	ATT-RK^18^			0.155	0.323	0.369	0.226	0.151	0.108
	CDGPT2^37^			0.257	0.289	0.387	0.245	0.166	0.111
	Original SAT^15^			0.320	0.361	0.433	0.281	0.194	0.138
	SAT$\dagger$			0.699	0.413	0.407	0.258	0.210	0.125
	Approach 1			0.687	0.402	0.450	0.299	0.224	0.141
	**Approach 2**			**0.701**	**0.450**	**0.520**	**0.390**	0.296	0.235
MS-COCO	BRNN^66^			–	–	0.642	0.451	0.304	0.203
	Original SAT^15^			–	–	0.718	0.504	0.357	0.250
	SAT$\dagger$			1.300	0.592	0.815	0.663	0.516	0.395
	Approach 1			1.298	0.505	0.818	0.664	0.509	0.385
	**Approach 2**			**1.360**	**0.606**	**0.821**	**0.672**	**0.529**	**0.409**

**Table 2:** The clinical efficacy (CE) metrics on the MIMIC-CXR dataset. The best results are highlighted in bold. Models labelled with $\dagger$ stand for the models we implemented and trained with the preprocessed MIMIC-CXR data. Other results are cited from the original papers. .

Model	Accuracy	Precision	Recall	F1-Score
S &T^12^	0.423	0.084	0.066	0.072
Original SAT^15^	0.703	0.181	0.134	0.144
TieNet^19^$\dagger$	0.741	0.265	0.178	0.197
NLG^29^$\dagger$	0.792	0.413	0.286	0.317
SAT$\dagger$	0.743	0.166	0.121	0.129
Approach 1	0.840	0.420	0.303	0.134
**Approach 2**	**0.861**	**0.445**	**0.351**	**0.369**

### Discussion

The first language model (SAT) learned to generate a short summary at the beginning of the report, based on the findings from a given medical image to provide the content details. This offers text generation direction seed for the second model. The preprocessing of the medical reports enabled these high metrics. We also address the biased data problem by applying domain-specific text preprocessing while using the NegBio labeller. In a radiology database, the data is unbalanced because abnormal cases are rarer than the normal ones. The NegBio labeller allowed us to get a not negative-biased diagnosis clinical records as it added short sentences at the beginning of the ground truth reports, making this task closer (in some ways) to a classification task, when the state-of-the-art models had already managed to achieve a strong performance. The SAT also provides 2D localization heatmaps of pathologies, assisting and accelerating the diagnosis process.

The second language model, the Generative Pretrained Transformer GPT-3, showed promising results in the medical domain. It successfully continued the extracted texts from the first language model, taking into consideration all the findings provided. As GPT-3 is a rather powerful transformer, it summarizes and provides more details on the findings. Natural language generation metrics suggest that using two language models subsequently provides a notable advantage. Such an approach can be considered as accurate and efficient for the medical captions generation.

One may notice a gap in the context-related performance (CIDEr)as each ground truth image is accompanied by multiple reference captions. The drawback in the CIDEr performance points to a suboptimal suitability of the generated output, whereas the Approach 2 does its best. This is due to the image-relevant n-grams occurring frequently in the respective set of reference sentences. The drawback is in the sampling from the GPT-3 distribution. The Approach 2, featuring SAT followed by the GPT-3, outperformed the reported state-of-the-art (SOTA) models in all the 3 datasets considered. Notably, the proposed approach outperforms SOTA models on MIMIC-CXR, demonstrating the highest performance in all the metrics. The performance for the main evaluation dataset, the MIMIC-CXR, is measured by the CE metrics using micro-averaging and demonstrates 0.861 for the proposed SAT + GPT-3 Approach 2 model vs. 0.840 with the Approach 1, and 0.743 for the SAT, respectively, as reported in Table 2.

Examples of the reports generated jointly via the SAT + GPT-3 with Approaches 1 and 2 are shown in Fig. 4. One may notice that some generated sentences coinside with the ground truth. For example, in both generated and the true reports, for the first X-ray it reads “no acute cardiopulmonary abnormality”. Some sentences are close in their meaning, even if they are different in terms of chosen words and n-grams (“no pneumonia. no pleural effusion. no edema. ...” compared to “ without pulmonary edema or pneumothorax”).

## Conclusions

We introduced a new technique of combining two language models for the medical image captioning task. Principally, the new preprocessing and squeezing approaches for clinical records were implemented along with a combined language model, where the first component is based on attention mechanism and the second one represents a generative pretrained transformer. The proposed combination of the models generates a descriptive textual summary with essential information on found pathologies along with their location and severity. Besides, the 2D Grad-CAM^67^ heatmaps localize each pathology on the original scans. The results, measured with the natural language generation metrics on both the MIMIC-CXR and the Open-I datasets, speak for an efficient applicability to the chest X-ray image captioning task. This approach also provides well-interpretable results and allows to support clinical decision making.

We investigated various approaches to automatic generation of X-ray image captioning. We proved that the SAT is a strong baseline, outperforming models with Transformer-based decoders. With the help of GPT-3 pre-trained language model, we managed to improve this baseline. The simple method, where the GPT-3 model finishes the report extracted by the Show-Attend-Tell model, yields significant improvements to the standard text generation scores. Recent advancements in interactive training, such as active learning^68^ and dialog-based ChatGPT^69^, have the potential to improve the performance of medical image captioning models even further. This is an area of research that will be explored in the future.

## Data Availability

All data generated or analysed during this study are included in this published article. The datasets used and/or analysed during the current study available from the corresponding author on reasonable request.
